# Subfoveal perfluorocarbon liquid removal by peeling of internal limiting membrane, without retinotomy

**DOI:** 10.5935/0004-2749.20200056

**Published:** 2020

**Authors:** Fernanda Silvestre, Gabriela Silvestre, Rodrigo Pessoa Cavalcanti Lira

**Affiliations:** 1 Universidade Federal de Pernambuco, Recife, PE, Brazil; 2 Universidade de Pernambuco, Recife, PE, Brazil

**Keywords:** Retinal detachments, Vitrectomy, Vitreoretinal surgery, Fluorocarbons, Intraoperative complications, Tomography, optical coherence, Descolamento retiniano, Vitrectomia, Cirurgia vitreorretiniana, Fluorcarbonetos, Complicações intraoperatórias, Tomografia de coerência óptica

## Abstract

Perfluorocarbon liquid has been widely used during vitreoretinal operations. Subretinal retention is a rare intraoperative complication, but even small quantities can damage the macular structure and function, and no standard treatment has been established. We encountered a 43-year-old woman who presented a retained subfoveal bubble after a vitreoretinal operation due to primary rhegmatogenous retinal detachment. Herein, we describe the procedure we used to remove the perfluorocarbon liquid without retinotomy using internal limiting membrane peeling.

## Abstract (pt)

O perfluorocarbono líquido tem sido amplamente durante cirurgias vitreorretinianas. A retenção subretiniana, é uma complicação intraoperatória rara, mas mesmo pequenas quantidades podem danificar a estrutura e função macular, e nenhum tratamento padrão foi estabelecido. Encontramos uma mulher de 43 anos que apresentou bolha subfoveal retida após uma cirurgia vitreorretiniana devido a descolamento de retina regmatogênico. Aqui, descrevemos o procedimento que usamos para remover o líquido perfluorocarbono sem retinotomia usando peeling da membrana limitante interna.

## INTRODUCTION

Perfluorocarbon liquid (PFCL) has been widely used during vitreoretinal operations^(1)^. Subretinal PFCL retention is a rare intraoperative complication, but even small quantities can damage the macula^(2)^. Visual acuity may improve following removal or displacement of subfoveal PFCL^(2)^. No standard method to treat this complication has been established. Subfoveal PFCL can be aspirated directly by retinotomy using a small gauge needle at the edge of^(3)^ or above^(4)^ the PFCL bubble. Other options include displacing the PFCL to the outside of the subfoveal space, followed by removal^(5)^ or displacement without removal^(6)^. Herein, we removed the subfoveal PFCL without retinotomy using only internal limiting membrane (ILM) peeling.

## CASE REPORT

**Figure 1:**
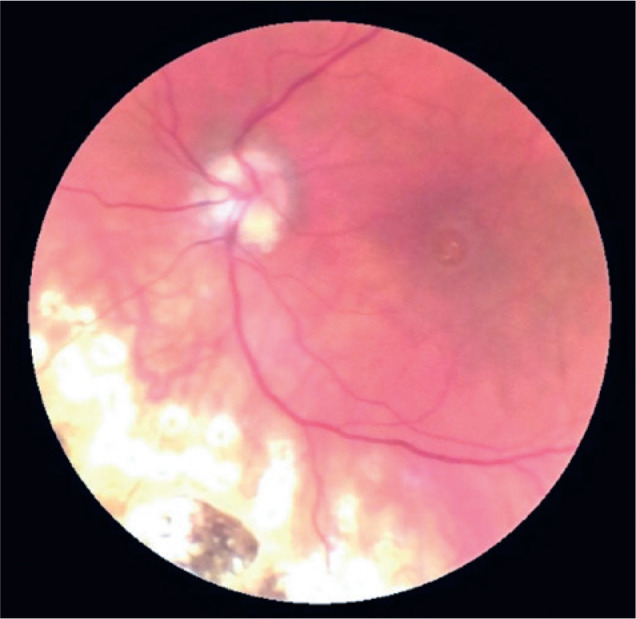
Photograph showing the fundus of the left eye with a macular pseudo-hole due to a retained subfoveal PFCL bubble.

**Figure 2:**
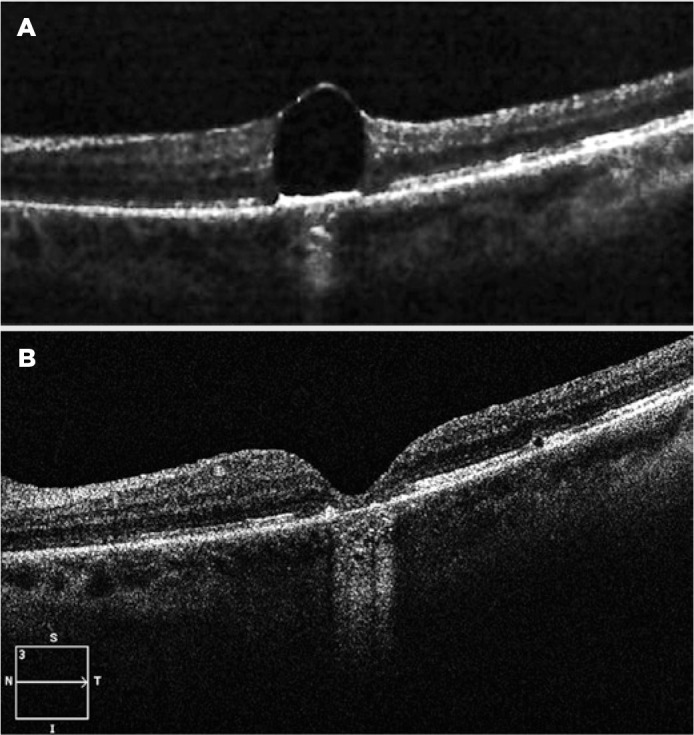
Preoperative and postoperative optical coherence tomography (OCT) demonstrating the removal of the subfoveal perfluorocarbon liquid (PFCL). A) Retained subfoveal PFCL bubble. B) Thinning, gliosis, and disruption of the external retinal layers in the foveal region on postoperative OCT.

A 43-year-old woman was seen in August 2017 with a suspected macular hole in her left eye (LE). Two months before that, her vision in that eye had deteriorated due to macula-off retinal detachment with an inferior retinal tear. Vitrectomy, PFCL injection, endolaser, and fluid-gas exchange had been performed in another institute. Her medical history included systemic arterial hypertension, bilateral optic disc drusen, bilateral LASIK surgery in 2011 (myopia of 6 diopters in both eyes), bilateral cataract surgery with insertion of intraocular lenses in 2013, YAG laser capsulotomy in 2014, and posterior vitreous detachment in 2016. The best-corrected visual acuity of the LE was 0.05, with metamorphopsia and central scotoma in the Amsler grid. Biomicroscopy, color vision test, and tonometry were all normal. We identified photocoagulation marks up to the lower border of the eye, slight pallor of the papilla, and a macular pseudo-hole due to a retained subfoveal PFCL bubble by ophthalmoscopy (Figure 1), although Watzke-Allen and laser aiming beam tests were positive. Optical coherence tomography (OCT) confirmed the diagnosis (Figure 2A).

The patient was scheduled to undergo a 25-gauge pars plana vitrectomy (Alcon Constellation Vision System, Fort Worth, Texas, USA). The surgeon stained the retinal surface with brilliant blue (Opht-Blue, Ophthalmos Rohto, São Paulo, Brazil), and removed the ILM in the foveal and temporal juxtafoveal region using forceps (Grieshaber^®^ asymmetrical forceps, Forth Worth, Texas, USA). The subfoveal PFCL bubble migrated spontaneously to the vitreous cavity through the foveal area where the ILM had been removed. The surgeon then aspirated the PFCL using a 41-gauge cannula (DORC, Zuidland, The Netherlands), and performed fluid-air exchange with 15% sulphur hexafluoride (SF_6_) gas tamponade. The patient was instructed to remain face down for a week. Six months following the operation, the patient’s visual acuity was 0.2, without metamorphopsia or central scotoma. Postoperative fundoscopy and OCT showed no macular holes; however, thinning, gliosis, and disruption of the external retinal layers in the foveal region were apparent (Figure 2B).

## DISCUSSION

We describe the case of a patient with retained subfoveal PFCL treated surgically by ILM removal without retinotomy. Her visual acuity improved; however, the visual prognosis was limited by characteristics associated with the previous retinal detachment surgery, and the location, size, and duration of the subfoveal PFCL. Moreover, we cannot rule out the possibility of an iatrogenic lesion during the removal^(2)^. However, real-time intraoperative OCT could render this surgical technique safer.

Direct surgical aspirations through a foveal or juxtafoveal retinotomy at the edge of the PFCL bubble have already been attempted, with varying results^(3,4)^. However, this procedure can cause sight-threatening complications, including macular holes, submacular hemorrhages, enlargement of the juxtafoveal retinotomy, or damage to the macular photoreceptor or retinal pigment epithelia. Because of these potential complications, a temporary retinal detachment was developed as a means of dis placing the retained subfoveal PFCLs^(5,6)^.

Our technique provides several advantages. The ILM covering the PFCL bubble is removed, leaving virtually no lesions or additional tissue losses; moreover, no macular retinal detachment or any additional damage to perifoveal photoreceptors are needed. Finally, this method is relatively simple to perform, requiring no special subretinal instruments.
